# How did the context of COVID-19 affect the implementation and mechanisms of participatory learning and action to address type 2 diabetes? Mixed-methods research in rural Bangladesh

**DOI:** 10.1136/bmjopen-2024-089288

**Published:** 2025-04-03

**Authors:** Joanna Morrison, Malini Pires, Sarker Ashraf Uddin Ahmed, Carina King, Tasnova Jerin Jeny, Raduan Hossin, Tasmin Nahar, Naveed Ahmed, Sanjit Shaha, Hassan Haghparast-Bidgoli, Abdul Kuddus, Kishwar Azad, Edward Fottrell

**Affiliations:** 1 Institute for Global Health, University College London, London, UK; 2 Centre for Health Research and Implementation, Diabetic Association of Bangladesh, Dhaka, Bangladesh; 3 Karolinska Institute, Stockholm, Stockholm, Sweden

**Keywords:** COVID-19, QUALITATIVE RESEARCH, Diabetes Mellitus, Type 2, PUBLIC HEALTH

## Abstract

**Abstract:**

**Objectives:**

Research indicates the effectiveness of participatory interventions to address rapid rises in type 2 diabetes in low-income countries. Understanding their transferability to different contexts is a priority. We aimed to analyse how the COVID-19 post-lockdown context and adjustments to a participatory learning and action intervention affected theorised mechanisms of effect in rural Bangladesh and to examine the broader implications of this context and intervention adjustments for developing optimal contexts for participatory interventions.

**Design:**

Mixed methods using longitudinal qualitative and quantitative observation data, focus group discussions and interviews with group and community members and project personnel. We used descriptive content analysis, guided by realist evaluation research questions about context, implementation and mechanisms. We used team reflection to enhance the rigour of our analysis.

**Setting:**

Cluster-randomised trial in Alfadanga upazila, Faridpur district, in the central region of Bangladesh. The intervention was implemented between January 2020 and December 2022, during the COVID-19 pandemic.

**Participants:**

Participatory group members, community members and project personnel (n=32). Structured observations of participatory groups (n=1820) and unstructured observations of groups and their environments (n=15).

**Interventions:**

Participatory learning and action community groups of men and women implemented by community-based facilitators.

**Results:**

Due to COVID-19, the participatory learning and action (PLA) intervention was not implemented as planned, which had major effects on the time available to develop the intervention with communities. Communities learnt about diabetes and were motivated to address its causes at an individual level, but community action was a more challenging mechanism to trigger. The post-pandemic context made it difficult to build community rapport, and strategies to engage communities through home visits were challenging. Communities’ prior negative experience in working together and in working with non-governmental organisations led to low community cohesion and low motivation to address diabetes collectively. This also resulted in expectations that the implementing organisation would implement community actions and incentivise attendance at meetings. This misalignment of expectations further disabled relationship building, and community strategies addressing the social causes of diabetes were largely not enacted.

**Conclusion:**

PLA has optimal effects when time is available to build trust and social cohesion. These are contextual elements and mechanisms that need to be activated to enable critical reflection and community action to develop an enabling environment to address type 2 diabetes.

**Trial registration number:**

ISRCTN42219712.

**STRENGTHS AND LIMITATIONS OF THIS STUDY**

- Our study engages participants and implementers of a participatory learning and action (PLA) intervention to understand the effect of the intervention and develop the theory about optimal contexts for PLA interventions.
- Through rigorous mixed methods and combining longitudinal data collection with data collected at the end of the intervention, we were able to improve understanding about the transferability of participatory learning and action to other contexts.
- We were limited in our ability to discuss the results of the cluster-randomised controlled trial evaluating the effect of the intervention with participants because data were unavailable at the time of this study.

## Introduction

 Diabetes remains an important public health issue. In 2021, there were around 529 million people with diabetes, and type 2 diabetes mellitus (T2DM) was responsible for 96% of this disease burden. Over three-quarters of those with T2DM live in low- and middle-income countries,^1^ and the T2DM burden is predicted to rise to 1.31 billion people by 2050.^2^ Social risk factors such as obesity, diet, environmental and occupational factors, tobacco, alcohol and physical inactivity contribute to increases in T2DM prevalence. Intervention approaches need to focus beyond the individual to understand how they interact with and are influenced by their social context. Building the capacity of communities to identify, mobilise and address social and structural risk factors is important to reduce the global T2DM burden.^3^

As part of a cluster-randomised trial, we implemented an intervention that sought to address T2DM through building community capacity using a participatory learning and action (PLA) cycle in rural Bangladesh. The intervention was implemented between January 2020 and December 2022, during the COVID-19 pandemic. We use mixed methods to describe and analyse how the COVID-19 context affected the implementation of the intervention and how this affected theorised PLA mechanisms of knowledge development, critical reflection and community action. Our paper adds to the literature about optimal contexts for PLA interventions and helps to explain the results of the now completed cluster-randomised controlled trial of the intervention (ISRCTN42219712).

### Implementation of the participatory learning and action intervention

In 2016–2017, an 18-month PLA intervention of community men’s and women’s meetings was effective in reducing the combined prevalence of T2DM and intermediate hyperglycaemia in rural communities in Faridpur, Bangladesh.^4^ In 2020, we sought to test the efficacy of the same intervention on a larger scale in a further cluster-randomised trial (ISRCTN42219712),^5^ but had to adapt the intervention because of the COVID-19 pandemic. The first national lockdown in Bangladesh was from 23 March to 30 May 2020, and restrictions continued until September 2020. We conducted a 1-month pilot in intervention areas in December 2020 and discussed the acceptability of implementing PLA with advisory groups and community members.^6^ Routine intervention implementation began in January 2021. A second lockdown from 5 April 2021 lasted only 2 weeks despite a high rise in cases between May and August 2021. We stopped all our activities during this time and resumed the intervention in November 2021.

The time for the intervention was shorter than planned, and we restricted group attendance to a maximum of 20 people. Members were invited to meetings after being phoned and screened for COVID-19 symptoms. Members were expected to wear masks, use hand sanitiser and sit at a distance from each other. Groups were convened by six male and six female facilitators who were local to the area, but not to every community. One had previous group facilitation experience and two had worked for non-governmental organisations (NGOs). They were given training about T2DM and community mobilisation, and a discussion manual. Facilitators were paid 9194 BDT per month (84 US$) and 7/12 worked until the end of the intervention. Two facilitation supervisors observed meetings, provided ongoing support and met facilitators every month.

**Figure 1:**
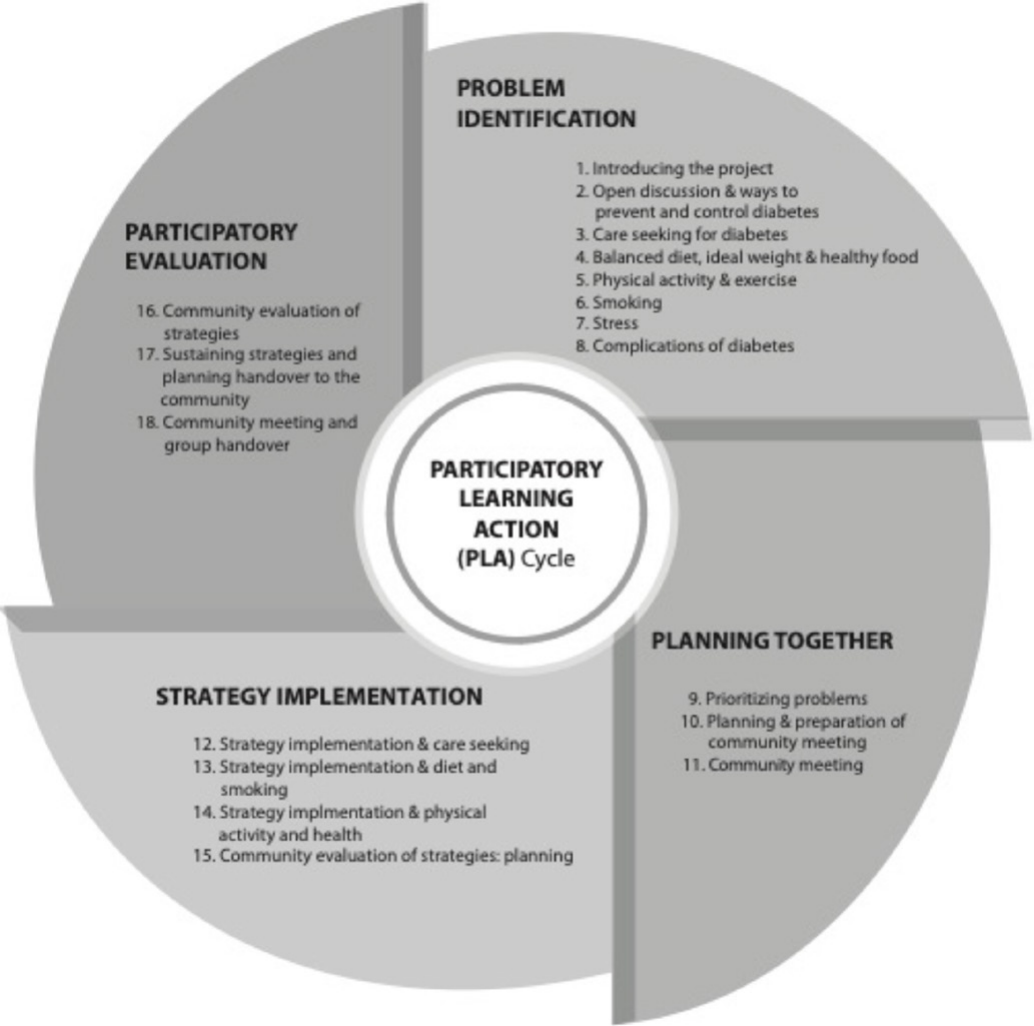
Participatory learning and action cycle.

Facilitators led groups through four phases of PLA (figure 1): (1) problem identification when participants identify and prioritise causes of T2DM and T2DM risk in their community (eight meetings); (2) planning together when groups and communities collectively design strategies to address the causes of T2DM that can be implemented by communities (two meetings); (3) strategy implementation of community-wide strategies (three meetings); (4) participatory evaluation of the strategies (two meetings).^7^ The shortened intervention period meant that several topics were discussed in each meeting as well as COVID-19 prevention, identification of signs and symptoms, and care-seeking behaviour. Groups were also encouraged to enact strategies in the problem identification phase, to accelerate action.

### Context, mechanisms and PLA

PLA is a complex, dynamic intervention and realist evaluation frameworks have been used to inform its evaluation.^8 9^ Realist evaluation considers that the delivery and components of the intervention interact with the context to produce mechanisms which lead to outcomes. Interventions can work through mechanisms of social change, where structures shape actions, which shape structures, in a continuing process of adaption.^10^ Interventions can also work through triggering mechanisms at the level of human reasoning.^11^ PLA is one such intervention that has been hypothesised to change health outcomes through social transformation as well as individual behavioural change. Our previous research about PLA emphasised the dynamic and synergistic relationship between the intervention creating enabled individuals, who could act within an enabled social context.^12^ In our current research, we explored how the context and implementation of PLA during COVID-19 may have affected theorised mechanisms and outcomes.

## Methods

### Setting

Alfadanga is a rural upazila of Faridpur district, south of Dhaka, on the Padma River. Faridpur has a population of over 1.7 million people living in 2000 square kilometres. Around 90% of the population are Muslim, and Hinduism is the second most populous religion.^13^ The main crops are jute and rice in this primarily agricultural economy. The district is prone to flooding, which can prevent access to healthcare and other facilities during the rainy season. In 2018, the prevalence of intermediate hyperglycaemia and T2DM was 17.2% and 8.9% among men and 23.4% and 11.5% among women, respectively.^14^ The Diabetic Association of Bangladesh hospital provides diabetes treatment in the district headquarters, and government community clinics and upazila health complexes should be able to screen for T2DM.

### Sampling and data collection

#### Data collection during the intervention (from January 2020 to December 2022)

To describe intervention implementation, facilitators and facilitation supervisors collected data about group participants—their age, the number who self-reported to have T2DM or family members with T2DM, and previous meeting attendance from all 108 PLA groups. They collected data on activities and adherence to COVID-19 procedures. They collected data on paper forms and entered these data into an ODK Collect app on Android devices in the office. Data were checked, errors corrected, and reported monthly to supervisors.

Process evaluation officers (RH & TJJ) conducted unstructured observations of 15 group meetings (table 1). No observations occurred between meetings 3 and 7, or after meeting 11 because of process evaluation personnel issues. They observed a cohort of three male groups, two female groups and one mixed gender group throughout the intervention. After meeting 3, the mixed gender groups were not observed as they had separated into men’s and women’s groups because men and women were not available at the same times of the day. Observation notes of meetings and conversations with community members were written in the community in Bangla, and the reports were made in English and shared for discussion with JM (a white, British, female senior researcher, with 15 years’ experience of working with teams in Bangladesh) and MP (a female researcher of mixed Portuguese and Indian origin with 5 years’ experience working on non-communicable diseases in South Asia).

**Table 1:** Observations

Gender of group	Community name	Meeting number & topic observed
Female	Uttar Pachuria	2 Open discussion about ways to prevent and control T2DM3 Care seeking for T2DM11 Community meeting
Female	Borobag	3 Care seeking for T2DM9 Prioritising problems11 Community meeting
Male	Dolairchor	2 Open discussion about ways to prevent and control T2DM3 Care seeking for T2DM9 Prioritising problems11 Community meeting
Male	Batepara	3 Care seeking for T2DM9 Prioritising problems11 Community meeting
Male and female mixed group	Charbakail	1 Introducing the project

T2DMtype 2 diabetes mellitus

#### Data collection after completion of the intervention (from December 2022 to February 2023)

We purposively sampled attenders of two men’s groups and two women’s groups which were run by different facilitators. Two groups were in a remote area. We sampled attenders to ensure a mix of ages, and some had T2DM. We purposively sampled the manager and the facilitation supervisors. We sought to sample all 12 male and female group facilitators, but we were unable to contact 5 facilitators as they had begun alternative employment. Therefore, we collected data from four female and three male facilitators (table 2).

**Table 2:** Data collection after completion of the intervention

Participant	Method	n (n participants)
Manager (participatory group)	Semistructured interview (SSI)	(1)
Facilitation supervisor (female)	SSI	1 (1)
Facilitation supervisor (male)	SSI	1 (1)
Facilitators (female)	Group interview (GI)	1 (2)
Facilitators (male)	GI	1 (3)
Facilitators (female)	SSI	2 (2)
Women’s group	Focus group discussion (FGD)	2 (12)
Men’s group	FGD	2 (11)
Total		10 (32)

Data were collected in Bangla by a trained male qualitative researcher (SAUA) who did not work on intervention implementation but was familiar with PLA. A facilitation supervisor and male facilitator located the homes of attenders and SAUA invited them to focus group discussions (FGDs). Four FGDs with five to six attenders were conducted in or around participants’ homes, and semistructured interviews (SSIs) with project personnel occurred in the project office or a place of convenience. JM conducted an SSI with the manager in English. Topic guides were used to discuss how COVID-19 had affected the intervention and context, experiences of attending and running groups, and factors affecting community action and participation of groups and communities in actions to address T2DM.

#### Data management and analysis

Observation and ODK data were tabulated and discussed every 2 months with process evaluation officers and researchers. A final report was made of these data. FGDs, group interviews and SSIs were recorded and directly translated into English for collaborative analysis. SAUA checked the quality of translation on a subsample of transcripts by comparing with the recording. Observation data and transcripts were imported into NVivo, and were read and discussed by MP, SAUA and JM over several online meetings. We conducted descriptive content analysis guided by our research questions: How did the community context affect interaction with the intervention?

How did COVID-19 affect community interaction with the intervention? How did COVID-19-related changes to the intervention affect interactions with the intervention? We created codes independently, discussed and then coded the data in NVivo. A mind map of the findings was presented to the wider team for discussion and validation. TN sought to emphasise a particular aspect of context, but largely there was agreement on our data interpretation.

To ensure anonymity, we have removed project personnel identifiers from quotations.

#### Patient and public involvement

Community members were not involved in setting the research question, design of methods, or dissemination plan, but they were intimately involved in the implementation of the intervention.

## Results

We describe the attendance and characteristics of the groups before presenting themes related to our three overarching research questions.

### Group characteristics

**Figure 2:**
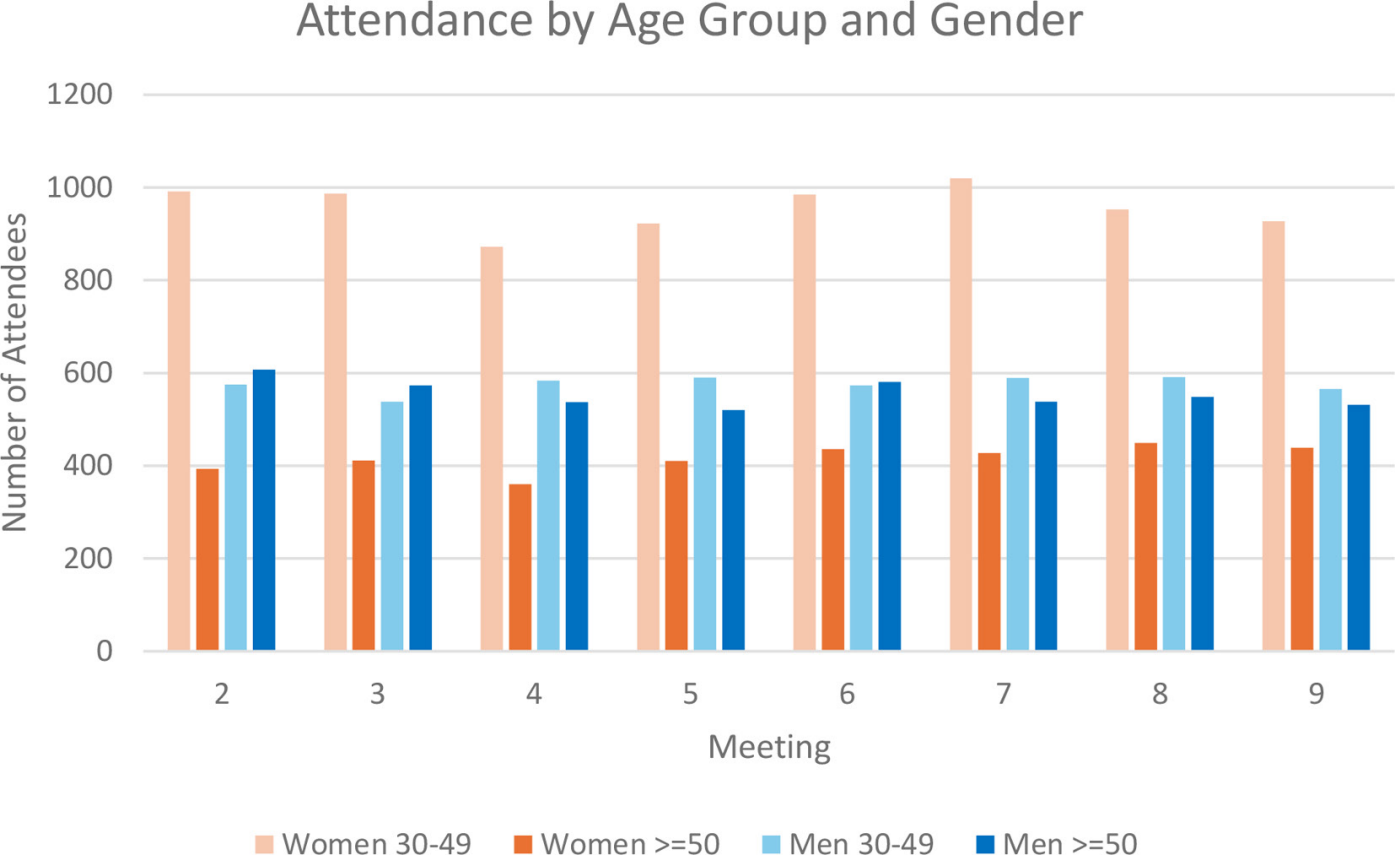
Attendance per meeting by age group and gender from intervention monitoring data.

Table 3 and figure 2 show group attendance from meetings 1–9. We do not have attendance records for meetings 10–13 because of process evaluation personnel issues. 10 groups were mixed gender but split after meeting 3 into separate men’s and women’s groups. Our data capture form did not register this change, and therefore mixed-group data is excluded where data are disaggregated by gender. On average, 13 participants attended meetings with attendance remaining constant over time. Women’s groups were slightly larger than men’s (14 vs 12 attenders per meeting) and had more young participants (69% aged 30–49) than men’s groups (50% aged 30–49). 12% of participants had family members with T2DM. 90% of women’s groups were conducted nearby a participant’s house and men’s groups tended to be in public places. Around 30% of men’s meetings were near a tea stall, or a shop.

**Table 3:** Attendance at meetings from intervention monitoring data (1 December 2020 to 2 March 2022)

		Total	Men’s groups	Women’s groups	Mixed groups
Meetings conducted (n)		1944			
Meetings with data extracted (n, %)		1820 (94%)	823	826	171
Total attendees at the meetings (n, %)		23 886	9743	11 853	2290
Average attendance (mean, range)		13.1 (5−40)	11.9 (5−40)	14.3 (7−33)	13.3 (9−19)
Attender age (n, %)	<30 years	88 (<1%)	51 (<1%)	22 (<1%)	15 (<1%)
	30–49 years	14 650 (61%)	4908 (50%)	8213 (69%)	1529 (67%)
	≥ 50 years	9148 (38%)	4784 (49%)	3618 (30%)	746 (32%)
Attender types (n, %)	Family members with T2DM	2911 (12%)	979 (10%)	1683 (14%)	249 (10%)
	Health workers	19 (<1%)	9 (<1%)	5 (<1%)	5 (<1%)

T2DMtype 2 diabetes mellitus

### How did COVID-19 affect community interaction with the intervention?

#### Residual fear and building trust

After COVID-19 restrictions were lifted, and we had consulted with community members, local advisory committees, politicians and our trial steering committee, we resumed group meetings. In some places, people were reluctant to attend: “People did not come to the meeting due to fear. A men’s group discussed: ‘We all knew that COVID-19 was a bad disease. It might spread by air. So why would I take a risk to go to the meeting?’ Many did not take this risk” (FGD). Project personnel noted how COVID-19 restrictions had affected their ability to build community rapport and encourage members to work together: “(after COVID-19) members said: ‘Do not come. Stay away. We will not go to the meeting and sit with you’… after some time and some discussion, they agreed. But they could not come to the meeting with a free mind and interact like before COVID-19, coming close together”. In a few places, community members were keen to restart the meetings, and this was easier when the project personnel were known to the community: “Members in all groups were not the same. In a few groups the members were very enthusiastic to hear the discussion. Furthermore, because I was the one running the meeting, and people knew me, people in my locality said ‘yes, let’s go to the meeting and listen’” (project personnel).

The COVID-19 control measures helped to build trust and some people came to the meeting to learn about COVID-19. Project personnel discussed: “We cleaned their hands with hand sanitizer, gave them masks and measured their temperature with the thermometer gun. As a result, they became interested. They understood that the project was on their side”. Members were interested in the new meeting format: “We were curious about the masks. These were new things. Members were sitting wearing them. We thought: ‘we will also take a mask and check our temperature’” (FGD women’s group).

### How did the COVID-19 adaptions affect the intervention?

#### Group size

Restricting group size was advantageous because social distancing was easier to maintain than in large groups and the discussion was more participatory: “Distance can be maintained if the meeting is done with fewer people, and everything you say can be heard. If there are more people, there is a situation of who will speak and who will listen?” (FGD women’s group). Members said that there were fewer disturbances from side-talk, and they found it easier to understand the discussions: “It is easy to understand in a small group, we can understand clearly, and the discussion is effective. In a large group some people become distracted” (FGD men’s group). Both project personnel and groups noted that splitting groups by location was more convenient for participants who could attend groups close to their homes, and it was often easier to find a meeting place for smaller groups: “When the group was large, there was problem to do the meeting. We had no place then. We had to conduct our meeting at the crossroads…it was a problem for vans and cars to get through” (FGD men’s group). Sometimes splitting the meeting enabled more people to come because they were more accessible than large groups, and sometimes local conflicts made some community members not want to participate in larger meetings with families they were not on good terms with: “There are social divisions. As a result, suppose I am a person from one clan (*gosti*) and a meeting has been called in my house. The opposite clan will say they will not come to my house. Running two meetings in two separate places was convenient for everyone” (project personnel).

#### Masks and social distancing

While members were keen to check their temperature and receive a free mask, all types of study participants agreed that masks prevented visual feedback which could stunt discussion, and many participants found masks uncomfortable. One project personnel said: “Many did not want to use them, they said: ‘we can’t breathe while wearing a mask, we cannot speak’”. The logistics of social distancing were challenging to manage, as multiple floor mats were required and often unavailable: “No one took out chairs, stools or *jolchowki* (low wooden stool) from their home. They thought if people sat on those, corona may spread” (project personnel). In addition, facilitators found it difficult to maintain limits on attendance and often members stood listening at the periphery.

#### Shortened timeline

Intervention implementation was delayed, disrupted and then resumed and this meant that discussion topics were combined in the adapted intervention. Process data show that meeting topics were covered as planned, but fewer groups used the participatory games. This affected the ability of members to discuss and understand risk factors. Project personnel said: “Discussing four topics in a month was a lot for members! It was difficult for them. We finished the meetings in a hurry because we had to complete them”.

#### Group-initiated strategies

Given our shortened timeline, facilitators encouraged group-initiated actions early in the intervention, instead of presenting and discussing strategies and actions at the community meeting after approximately 10 months. Group-initiated strategies were usually individual, not collective or community focused. Groups perceived the community meeting to be a dissemination programme, not a way to validate strategies and work with the broader community to address the causes of T2DM. Consequently, group attendance lessened after this meeting and only a few groups took actions in the remaining few months of the intervention.

Health promotion through home visits had been a common strategy in previous interventions but groups were reluctant to do this because of COVID-19. Some families did not allow members to enter households or to visit other households. In previous interventions, women often exercised in groups for safety and to help challenge gender norms. COVID-19 had added more barriers to physical activity for women: “Women cannot go for a walk. They feel shy or do not get time because they have a lot of work at home… Women thought if we go out and get infected by COVID-19, then the family will not be able to function because they would not be able to cook and feed husbands and male family members” (project personnel). Women feared being blamed for bringing COVID-19 into the family.

Fundraising among members to pay for T2DM-related expenses had been implemented in previous PLA interventions but was difficult to implement while there was uncertainty about COVID-19 and the status of the groups. Funds were made, then redistributed, and there was often a reluctance to restart. Previous experience with NGOs stealing funds was also a disincentive to fund raising: “Many asked ‘what is the benefit of doing this’? and doubted whether they will get back the money” (FGD women’s group). Project personnel also reported that COVID-19-related financial difficulties meant that many did not want to invest in a fund or have time to take collective action.

### How did the community context affect interaction with the intervention?

#### Lack of confidence in knowledge about how to prevent and control T2DM

Communities were generally interested in learning about how to prevent and control T2DM. A few did not attend meetings because they did not feel at risk, but most found the meetings useful, and said they had learnt from the discussions. This knowledge was disseminated to friends and neighbours, and to the wider community through the community meeting. Face-to-face interaction and learning from people with T2DM were appreciated: “Those who have diabetes, if they visit a doctor, they just give them a book. But how many people can read a book? Seeing something and hearing it directly from someone’s mouth is a more effective way of learning because people can remember more, and more people became aware because of the community meeting at the market area” (FGD women’s group). Women noted that they gained social support from the intervention as well as learning about T2DM: “When we met, one or two people who had diabetes shared their experience in the meeting. We benefitted from hearing the discussion about diabetes…We liked this. If someone’s mind is not well, meeting with five people can make her feel well” (FGD women’s group).

#### Norms, health systems weaknesses and vulnerabilities

Groups discussed the embarrassment and shame around enacting preventative behaviours, and harmful gender norms made it more difficult for women. An observation report of a women’s group described a discussion about physical activity: “We feel ashamed walking in front of villagers, we get no time after finishing household tasks. Our In-laws think exercise is an excuse for skipping work!” (observation women’s group). Many felt that embarrassment about going for a walk had reduced since the group had been meeting: “Before, I thought, ‘should I go for a walk?’ I was afraid in case someone would say something. Now there is no problem, and nobody says anything. I no longer feel fearful” (FGD women’s group). There was also social stigma attached to buying cheap vegetables in the market, and in doing behaviours that could reveal a T2DM diagnosis: “Before covid and before this meeting, many people wanted to hide their diabetes. Now many know about diabetes. So, what is the benefit of hiding it?” (FGD men’s group).

Community clinics did not routinely provide screening services, due to lack of blood glucose test strips and functional glucometers. Groups were reluctant to engage with clinics about this because they did not want to exacerbate issues of unequal treatment and thought their concerns would be ignored. Groups felt vulnerable if they complained at the upazila level: “(Members) found it difficult to talk to the chairman or Upazilla Health and Family Planning Officer regarding this issue. (Members) were afraid that the community clinic personnel might get angry if they complain and then they wouldn’t get other health services after that” (observation men’s group). The main barriers to care seeking at referral centres or private health facilities were financial and time poverty, and women found it difficult to find someone to accompany them.

#### Expectations

Project personnel said it was difficult to keep groups engaged because of the history of incentivised attendance at group meetings promoted by government and NGOs, and the expectation that project personnel would implement community strategies: “When we discussed about strategies and how to implement them, they weren’t interested. They thought: ‘Why have we been given these responsibilities on our shoulders?’” (project personnel). Project personnel were often expected to incentivise actions, for example, by providing small loans to implement kitchen gardening, and free or subsidised provision of blood glucose testing: “Most people here are poor. Whenever we asked them to test for diabetes, they told *us* to do the test” (project personnel). Women said: “Many came to the meeting because they were interested in testing for diabetes, but it was not done. (the implementing organisation) should do this” (FGD). Facilitators of 12 women’s group meetings bought blood pressure machines (sometimes with contributions from members) and measured blood pressure after the meeting, despite not receiving training on how to do this: “I used to measure (blood) pressure after the meeting. If there were 18 people at the meeting, more people would check their blood pressure. When they asked me to bring medicine from the office, I told them that the office would not give it for free. Then they said we will buy it if it is given them for a lower cost” (project personnel).

Project personnel noted that expectations of incentivised attendance at meetings extended into accusations of misappropriation of funds: “Members said: ‘some organisations give us food and money, and you give nothing.’ An idea was created in the community that money had been allocated for them, but we embezzled the money. But we overcame this challenge, and they understood that this organization delivers more important things than money” (project personnel). Female members discussed community perceptions of benefits to project personnel versus themselves which made them uncomfortable to attend: “Many made comments saying, ‘while we are going to the meeting the project personnel will get money, but what is our benefit?’ Many people complained that (project personnel) do not test for diabetes and instead of going to the meeting they do their own work” (FGD women’s group).

## Discussion

**Figure 3:**
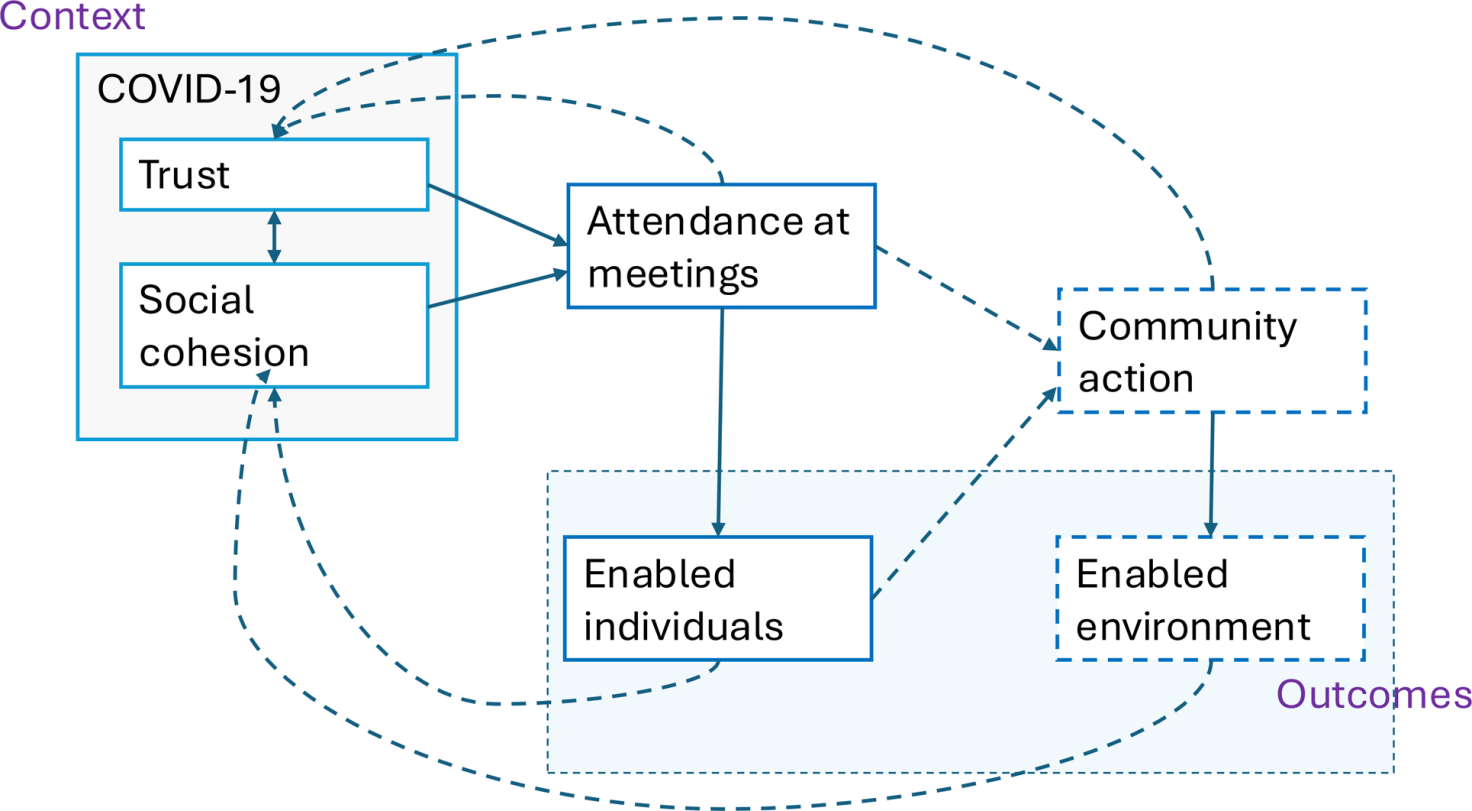
Context, (partially triggered) mechanisms and (partially achieved) outcomes.

We have described how the COVID-19 and existing community context affected the implementation of the intervention, and the community response. The intervention increased knowledge and awareness of T2DM but a pre-existing context of high expectations, mistrust of NGOs and poor quality of health services was a suboptimal environment for PLA. This context, combined with residual fear of COVID-19 and the shorter time frame made it more difficult to build trust, to build understanding about the method of the intervention, to develop a collective awareness about the community barriers to behaviour change, and for groups to initiate collective action (figure 3). These findings enable us to develop the theory around key contextual elements that trigger the intervention to be effective.

### Optimal contextual environments and delivery mechanisms for PLA

When analysing the complexity of context, it is useful to create an initial system description.^15^ This description can help evaluate the extent to which the intervention is aligned with the interests of those in intervention areas and identify what may prevent the intervention from being effective. Our previous research in Faridpur District meant we had an understanding about community perceptions of risk factors, management and prevention strategies for T2DM.^16^,^18^ It was clear from this research that a community focus and addressing social norms were necessary for intervention success. Our analysis of longitudinal and retrospective data has identified contextual elements that affected our intervention and contributes to theory about the contexts which can facilitate intervention success.

#### Cohesion

Social cohesion refers to the degree of social connectedness and solidarity between different community groups within a society, connectedness to place, and orientation towards the social good.^19^ Research suggests that communities with lower social cohesion pre-COVID-19 were less resilient, and people in those communities suffered from poorer mental health.^20^,^22^ Disasters such as COVID-19 can exacerbate pre-existing divisions and inequalities,^23^ and during the pandemic, the importance of social cohesion for health was recognised and promoted.^24 25^ Our data indicate that intervention areas were not all cohesive. There was some conflict between communities, and their prior experience with NGOs and savings funds had been divisive. This prior experience, the post-pandemic environment of suspicion of outsiders^6^ and social distancing measures made it challenging to build relationships between the intervention team and the community, and between community members. This may have resulted in a lack of understanding about the intervention method. Our study indicates that in communities where social cohesion is low, time and effort are necessary to develop this in a post-pandemic context.

#### Trust

Trust is a key contextual factor which can affect the success of participatory interventions.^26^ Particularly in the collectivist cultural context of rural South Asia, strong personal and informal relationships between project personnel and communities are important to build trust and develop a shared understanding of the intervention.^27^ When pre-existing trust based on prior positive experience or partnership has not been established, it can be a time-consuming and complex process to build relationships.

Previous experience with NGOs in our intervention area had created a context of mistrust, where NGOs were seen to benefit from interventions at the expense of communities, and the expectation of incentivised participation was unmet in our study. In addition, the implementing organisation did not provide health services, which communities expected, which may have further eroded relationships. This indicated that communities did not understand the nature of the intervention. Remuneration and incentivisation may enable the participation of the most marginalised, but it can also motivate participation for financial or reputational reasons which has the potential to erode trust between participants and researchers.^28^ We may have been more successful in building relationships if the team had been able to interact more with the community over time, using participatory games and methods that we had planned to use to align goals and expectations and explore the drivers of T2DM.^29^ Community participation in conceptualising and implementing strategies was minimal, and this participation is an important mechanism to create an enabled environment for behaviour change. Insufficient time to build a context of trust with the community affected the mechanisms of how PLA can address T2DM.

#### Attendance

Our data showed a somewhat stigmatising effect of group attendance or participation in group activities when no material incentives were given. Only 32% of those in the endline survey in intervention areas reported ever participating in groups, and only 16% participated in the community meeting.^30^ This attendance level is lower than in previous PLA interventions.^7^ Low repeat attendance and low participation in community meetings resulted in challenges to develop other key mechanisms, such as a shared understanding of the intervention, and the development of solidarity and unity to enact community change. A review of barriers and enablers for participatory interventions for maternal health has also shown that low meeting attendance can hinder community action.^31^

#### Shared understanding

The context which we have described made it challenging to develop collective understanding about the causes of T2DM. While community members were keen to learn from the intervention, and their knowledge of T2DM did increase, this did not translate to a critical analysis of the social determinants of T2DM. Without this critical analysis, there was less motivation or felt need for collective action. The community meeting is also an important time to develop a shared understanding. The low participation and understanding of this meeting as a ‘closing’ of the intervention also meant that this opportunity was not realised.

#### Community action

Previous PLA research has shown that planning together and community action components of the intervention are key in legitimising the group, changing norms and addressing the broader social issues affecting health.^32^,^34^ The successful development of solidarity through collective problem solving and community empowerment through implementing actions has also been identified as an enabling element to participatory non-communicable disease-focused interventions.^28^ We have shown that these key mechanisms were not triggered to the same extent as in previous interventions.

#### Study limitations

Data collection was interrupted by personnel changes, and by COVID-19 restrictions. We were unable to discuss the trial results with participants because data were unavailable at the time of this study. Some social desirability bias may have affected the data, but we tried to mitigate this somewhat by having endline data collected by a researcher unknown in the community.

## Conclusion

Our paper adds to the literature about optimal contexts and implementation strategies for PLA interventions and helps to explain the results of the cluster-randomised controlled trial research evaluating the intervention. Contextual and implementation factors affected PLA mechanisms of critical reflection and community action, which help to explain why the intervention increased knowledge and awareness of T2DM but did not enable broader community change. Our research demonstrates the importance of time to build and maintain community relationships and develop a collective understanding of the PLA approach. Without adequate time, it is challenging to build trust and enable collective action. Our study adds to the evidence that addressing the social and structural risk factors to enabling individual and community behaviour change are required to reduce the global T2DM burden.

## Data Availability

Data are available upon reasonable request.
