# Supplementary material for: How did the context of COVID-19 affect the implementation and mechanisms of participatory learning and action to address type 2 diabetes? Mixed-methods research in rural Bangladesh
Source: BMJ Open. 2025 Apr 3;15(4):e089288. doi: 10.1136/bmjopen-2024-089288 (PMC11969586; doi:10.1136/bmjopen-2024-089288)
Supplement: Supplementary file 1 [file bmjopen-2024-089288.reviewer_comments.pdf]

PEER REVIEW HISTORY

BMJ Open publishes all reviews undertaken for accepted manuscripts. Reviewers are asked to complete a checklist review form and are provided with free text boxes to elaborate on their assessment. These free text comments are reproduced below.

ARTICLE DETAILS

Title (Provisional)

How did the context of COVID-19 affect the implementation and mechanisms of participatory learning and action to address type 2 diabetes? Mixed methods research in rural Bangladesh

Authors

Morrison, Joanna; Pires, Malini; Ahmed, Sarker Ashraf Uddin; King, Carina; Jeny, Tasnova Jerin; Hossin, Raduan; Nahar, Tasmin; Ahmed, Naveed; Shaha, Sanjit; Haghparast-Bidgoli, Hassan; Kuddus, Abdul; Azad, Kishwar; Fottrell, Edward

---

VERSION 1 - REVIEW

**Reviewer**	**1**
**Name**	**Dack, Kyle**
**Affiliation**	**University of Bristol, MRC Integrative Epidemiology Unit**
**Date**	**10-Sep-2024**
**COI**	**None**

This paper evaluates participatory interventions for type 2 diabetes in Bangladesh during the Covid-19 pandemic. It is very well written, including details of the methodology and justifications for decisions made. I have only a few minor comments:

P2/Line 18-19. Please include the study period.

P3/Line 15. Is there a reference for 75% of T2DM prevalence being in low/middle income countries? If it the same as the previous sentence it may be clearer to join the two sentences and move the reference to the end.

P3/Line 32. It looks like this may be a currently ongoing trial? If so it would be good to add this.

P3/Line 44. This line states the intervention started in November 2021. However, line 26 states it was implemented starting in January 2020. Table 1 includes intervention monitoring data from “1st December 2020”. It is currently difficult to see how these all fit together, and a brief explanation of the timeline may be helpful somewhere in the methods.

**Reviewer**	**2**
**Name**	**Hashim, Jumana**
**Affiliation**	**National University of Singapore, School of Medicine**
**Date**	**31-Jan-2025**
**COI**	**NA**

The paper aims to explore an important topic and bring forward a timely discussion. The paper could be considered with some revisions. Some suggestions are below:

1. The manuscript is slightly hard to follow as the research questions are actually about the participant perception due to COVID-19 and not the actual PLA intervention

a. So this then bodes the question of why we needed to talk about the intervention and/or t2d management/prevention – if the context of PLA is important then some description of the PLA intervention is necessary to set the scene.

b. The PPI section alludes to the implementation of the intervention by the participants, but the intervention itself is not described so perhaps this part is not required?

c. Perhaps the paper can be streamlined to just focus on the research question regarding the impact of COVID-19?

2. The tables are confusing as the rows and columns are hard to follow. For example:

a. What does the meeting observed column in Table 1 mean? And why does it matter?

b. Table 2 is not clear as to what it is describing – what does the last column mean – and how does it impact your findings?

c. Table 3 row labels are not fitting to what they are reporting – for example the first two rows I believe are reporting the number of participants and not the number of meetings? But that might also not be true as I don't get the sense that that many participants were involved. Additionally, the different number from the different age groups do not add up to the total number of participants.

3. The results from the study is rich and can benefit from being further organized into sub themes to provide more clarity and highlight the different topics within each theme.

4. Some of the manuscript, especially the discussion feel repeated – consider streamlining and being concise.

VERSION 1 - AUTHOR RESPONSE

Reviewer: 1

Dr. Kyle Dack, University of Bristol

Comments to the Author:

This paper evaluates participatory interventions for type 2 diabetes in Bangladesh during the Covid-19 pandemic. It is very well written, including details of the methodology and justifications for decisions made. I have only a few minor comments:

We thank the reviewer for their comment.

P2/Line 18-19. Please include the study period.

We have revised the abstract as requested.

P3/Line 15. Is there a reference for 75% of T2DM prevalence being in low/middle income countries? If it the same as the previous sentence it may be clearer to join the two sentences and move the reference to the end.

We have revised this text and added a reference.

P3/Line 32. It looks like this may be a currently ongoing trial? If so it would be good to add this.

The trial has been completed and the trial manuscript is under review. We have added this to the text.

P3/Line 44. This line states the intervention started in November 2021. However, line 26 states it was implemented starting in January 2020. Table 1 includes intervention monitoring data from “1st December 2020”. It is currently difficult to see how these all fit together, and a brief explanation of the timeline may be helpful somewhere in the methods.

We apologise for this confusion. We have added to the text at the bottom of page 2 to clarify the timeline.

Reviewer: 2

Dr. Jumana Hashim, National University of Singapore

Comments to the Author:

The paper aims to explore an important topic and bring forward a timely discussion. The paper could be considered with some revisions. Some suggestions are below:

1. The manuscript is slightly hard to follow as the research questions are actually about the participant perception due to COVID-19 and not the actual PLA intervention

a. So this then bodes the question of why we needed to talk about the intervention and/or t2d management/prevention – if the context of PLA is important then some description of the PLA intervention is necessary to set the scene.

b. The PPI section alludes to the implementation of the intervention by the participants, but the intervention itself is not described so perhaps this part is not required?

We thank the reviewer for their comment. We have added a Figure to help describe the PLA intervention on page 3 and provided a reference to literature which describes the implementation of PLA for Type 2 diabetes in detail.

c. Perhaps the paper can be streamlined to just focus on the research question regarding the impact of COVID-19?

The Medical Research Council guidance on process evaluations emphasises the need for complex interventions to be examined within their contexts as this can affect the transferability of interventions to other settings, and can influence the triggering of key mechanisms which make the intervention effective. Our manuscript is a process evaluation of the intervention. We analyse how the PLA intervention works or does not work in this context. An important part of the context is the COVID-19 pandemic and strict lockdowns. Therefore the focus of the research is about the PLA intervention in the context.

2. The tables are confusing as the rows and columns are hard to follow. For example:

a. What does the meeting observed column in Table 1 mean? And why does it matter?

We observed meetings which had different topics. The topics are referred to in the Figure, and we have added description to the table. It is important to report this so the reader can see that we were rigorous in planning and implementing observation data over time, and observing different meeting topics revealed different findings about groups and the way that they worked.

b. Table 2 is not clear as to what it is describing – what does the last column mean – and how does it impact your findings?

Table 2 describes the participants in the qualitative data collection. The last column refers to the number of participants who participated in interviews, group interviews and focus group discussions. These are not the participants in the intervention (ie community group members), but a purposive sample with whom we collected data focused on their experience of the intervention and the context. The number of participants in this study was sufficient to generate theoretical generalisable results.

c. Table 3 row labels are not fitting to what they are reporting – for example the first two rows I believe are reporting the number of participants and not the number of meetings? But that might also not be true as I don't get the sense that that many participants were involved. Additionally, the different number from the different age groups do not add up to the total number of participants.

The first two rows are reporting on the number of meetings over the study period. On page 6 in the manuscript we state: on average 13 participants attended meetings, with attendance remaining constant over time. We have updated the numbers in the table – thank you for noticing this error, we had not updated the table after doing some additional data checks.

3. The results from the study is rich and can benefit from being further organized into sub themes to provide more clarity and highlight the different topics within each theme.

We thank the reviewer for this comment, however we only have 1,2, or 3 paragraphs under each theme, and think that splitting the data further into sub themes would detract from our findings.

4. Some of the manuscript, especially the discussion feel repeated – consider streamlining and being concise.

We thank the reviewer for their comment and have consolidated the discussion section.

VERSION 2 - REVIEW

**Reviewer**	**2**
**Name**	**Hashim, Jumana**
**Affiliation**	**National University of Singapore, School of Medicine**
**Date**	**18-Mar-2025**
**COI**	

The authors have addressed the comments meaningfully
